# Effect of cAMP Signaling Regulation in Osteogenic Differentiation of Adipose-Derived Mesenchymal Stem Cells

**DOI:** 10.3390/cells9071587

**Published:** 2020-06-30

**Authors:** Sławomir Rumiński, Ilona Kalaszczyńska, Małgorzata Lewandowska-Szumieł

**Affiliations:** 1 Department of Histology and Embryology, Center for Biostructure Research, Medical University of Warsaw, 02-004 Warsaw, Poland; slawomir.ruminski@wum.edu.pl; 2 Postgraduate School of Molecular Medicine, Medical University of Warsaw, 02-091 Warsaw, Poland; 3 Laboratory for Cell Research and Application, Medical University of Warsaw, 02-097 Warsaw, Poland

**Keywords:** osteogenic differentiation, cAMP, mesenchymal stem cells

## Abstract

The successful implementation of adipose-derived mesenchymal stem cells (ADSCs) in bone regeneration depends on efficient osteogenic differentiation. However, a literature survey and our own experience demonstrated that current differentiation methods are not effective enough. Since the differentiation of mesenchymal stem cells (MSCs) into osteoblasts and adipocytes can be regulated by cyclic adenosine monophosphate (cAMP) signaling, we investigated the effects of cAMP activator, forskolin, and inhibitor, SQ 22,536, on the early and late osteogenic differentiation of ADSCs cultured in spheroids or in a monolayer. Intracellular cAMP concentration, protein kinase A (PKA) activity, and inhibitor *of DNA* binding 2 (*ID2*) expression examination confirmed cAMP up- and downregulation. cAMP upregulation inhibited the cell cycle and protected ADSCs from osteogenic medium (OM)-induced apoptosis. Surprisingly, the upregulation of cAMP level at the early stages of osteogenic differentiation downregulated the expression of osteogenic markers *RUNX2*, *Osterix*, and *IBSP*, which was more significant in spheroids, and it is used for the more efficient commitment of ADSCs into preosteoblasts, according to the previously reported protocol. However, cAMP upregulation in a culture of ADSCs in spheroids resulted in significantly increased osteocalcin production and mineralization. Thus, undifferentiated and predifferentiated ADSCs respond differently to cAMP pathway stimulation in terms of osteogenesis, which might explain the ambiguous results from the literature.

## 1. Introduction

Adipose-derived mesenchymal stem cells (ADSCs) are a subtype of mesenchymal stem cells (MSCs) that reside in the stromal vascular fraction (SVF) of adipose tissue. Both crude SVF and cultured ADSCs are being extensively studied in preclinical and clinical trials, aiming to provide a tool for the treatment of a number of human disorders. These disorders include soft-tissue defects, cardiovascular diseases, neurological impairments, autoimmunological diseases, and skeletal defects [1].

ADSC have several important characteristics from the point of view of their applicability in bone-regeneration therapies. First, ADSCs secrete an array of soluble factors, such as vascular endothelial growth factor (VEGF), platelet-derived growth factor (PDGF), hepatocyte growth factor (HGF), bone morphogenetic protein 2 (BMP2), and matrix metalloproteinases that stimulate neovascularization, wound healing, matrix remodeling, and osteoblast-precursor differentiation. Second, the capacity of ADSCs to differentiate into osteogenic lineage cells is postulated to provide the direct replacement or augmentation of resident-osteoblast functions after ADSC transplantation, thus allowing the production of a mineralized bone matrix at the defect site [2,3].

The most often postulated mechanism of MSCs’ regenerative action relays on trophic factors secreted by the introduced cells, which stimulate resident progenitor cells to undertake physiological regenerative processes. The probability that the transplanted MSCs form new tissue themselves is low [4]. Experiment data from in vivo animal studies support this view, as ADSCs showed poor survival after in vivo ectopic implantation [5]. In addition, a study performed by Ando et al. on a mouse high-speed distraction osteogenesis model proved that bone marrow-derived mesenchymal stem cells (BMSCs) injected into a bone gap induced bone callus formation in the gap, which is otherwise filled with fibrotic tissue in the control animals. However, the injected BMSCs were not detectable at the implantation site after 10 days. This study also reported equally effective bone callus formation in experiment animals treated with BMSCs as that with BMSC-conditioned medium [6], which indicated that cell-derived secreted factors were responsible for callus formation.

Results of in vivo studies using ADSCs, as presented above, also suggest a paracrine mechanism of bone regeneration. When residual osteogenic precursors are present at the implantation site, bone healing occurs, but in the case that these precursor cells are missing (i.e., subcutaneous implantation was performed), bone formation is inefficient.

The success of the direct replacement approach is at least partially hampered by the relatively low efficiency of osteogenic differentiation of ADSCs in vitro [7,8]. Furthermore, bone-forming capacity in vivo, as evidenced by the subcutaneous implantation of cell-seeded hydroxylapatite (HA) discs in a rat model, is low compared to the capacity of bone marrow MSCs (BMSCs) [9]. Furthermore, our study showed that in vitro predifferentiated ADSCs implanted on a polycaprolactone-based scaffold did not result in new bone formation after subcutaneous implantation in mice [10].

Therefore, the osteogenic-differentiation process of MSCs, and ADSCs in particular, needs to be studied further, with the aim to develop an improved method of bone-forming cells in vitro and in vivo.

Differentiation of MSCs into osteoblasts is regulated by several signaling pathways. These pathways include the Wnt/beta-catenin [11,12], extracellular signal-regulated protein kinase 1/2 (Erk1/2) [13,14], and cAMP signaling pathways [15], Hedgehog signaling [16], and bone-morphogenic protein [17,18]. However, in most of the studies aiming to elucidate the molecular mechanisms of therapeutic mesenchymal stem cells (MSC) capabilities, BMSCs or murine MSC cell lines were used. Data on the mechanisms of ADSC osteogenesis are relatively scarce.

cAMP signaling is particularly interesting in the context of ADSC osteogenic differentiation. Signaling is triggered by the activation of G-coupled receptor proteins (GPCRs) that induce cAMP synthesis by adenylate cyclase. The physiological ligands that activate parathyroid hormone 1 receptor (PTH1R), the G protein-coupled receptor in osteoblasts, are parathormone (PTH) and parathormone-related protein (PTHrP) [19,20]. The produced cAMP activates protein kinase A (PKA) that can then phosphorylate the cyclic–AMP response element binding protein (CREB). Phosphorylated CREB binds to the CREB response element (CRE) in the DNA to activate transcription of the target genes [15]. Most of the published data indicate that BMSC osteogenic differentiation is stimulated by the activation of the cAMP signaling pathway, and phosphorylated CREB induces the expression of osteoblast characteristic genes [15,21,22,23]. However, several authors also reported that cAMP signaling-pathway activators enhance adipogenesis [24] or even cause a switch in lineage commitment from osteoblasts to adipocytes, as studied on BMSCs cultured in osteogenic medium before inducing adipogenesis [25]. In the case of the latter study, the unexpected cellular response upon PKA activation may be partly explained by the high concentration (10–7 M) of dexamethasone used in the osteogenic medium applied there. Furthermore, Doorn and coauthors pointed out that two different cAMP analogs that activate PKA, db-cAMP, and 8-br-cAMP induce either osteogenesis or adipogenesis, respectively [26].

Furthermore, data on the role of cAMP signaling in the maintenance of balance between osteogenesis and adipogenesis in adipose-tissue cells seem to add even more complexity to the whole process. Human genetic diseases, such as progressive osseous heteroplasia (POH), are caused by inactivating mutations in *GNAS* encoding the α-subunit of the stimulatory G protein (Gsα) that abrogate the activation of adenylate cyclase. On the animal model, Gsα+/− knock-out mice, POH was manifested by ectopic bone formation in several tissue types, including adipose tissue that stems from abnormally high osteogenic activity of mesenchymal precursors residing in the adipose tissue [27]. A similar mechanism was observed in murine-cultured ADSC, carrying a heterozygous knock-out in *Gsα*. Mineralization, alkaline phosphatase (ALP) activity, and the expression of *SPP1* (osteopontin) and *OCN* (osteocalcin) genes were upregulated in knock-out versus wild-type ADSCs while cultured in the osteogenic medium [28]. A subsequent study found that the mutation was also associated with the inhibited adipogenic differentiation of murine ADSC, which indicated that the perturbation of cAMP signaling pushes the balance in favor of osteogenesis [29]. However, a recent study on rat ADSCs showed that activation of the cAMP pathway by zinc ions and an electromagnetic field resulted in the upregulation of ALP activity and the expression of *RUNX2*, *OCN*, and *BMP2* genes [30]. The contradictory results obtained by distinct groups might be explained by interspecies variations or by different cAMP stimulants used. Nonetheless, the role of cAMP pathway activation on the differentiation of ADSCs into the osteogenic lineage in vitro remains unclear.

In addition to pro-osteogenic biochemical inducers present in the culture medium, the dimensionality and architecture of the culture system might also play a role in the osteogenic differentiation of ADSCs. The expression of several osteogenic lineage characteristic genes was found to be upregulated in the three-dimensional (3D) spheroid culture system compared to a traditional two-dimensional (2D) culture [31,32]. The precise mechanism is not clear, but enhanced cell-to-cell communication [33] and cell-to-extracellular-matrix (ECM) signaling [34] were found to play a role. Our former studies on ADSC osteogenesis indicated that both biodegradable 3D scaffolds based on poly(epsilon-caprolactone [10] and 3D scaffold-free multicellular spheroids [35] enhance osteogenic differentiation.

Here, we aimed to study the effect of cAMP regulation on the osteogenic differentiation of ADSCs using a soluble activator (forskolin, FSK) and inhibitor (SQ 22,536) of adenylate cyclase. In addition to the standard 2D culture, we employed a 3D spheroid culture to provide improved osteogenic stimulation and analyze the possible role of PKA activity in 3D-induced osteogenesis. Human ADSCs were used as a study model in order to provide experiment data that might be valuable for the therapeutic application of autologous cells in skeletal disorders.

## 2. Materials and Methods

### 2.1. Adipose-Derived Stem-Cell Isolation and Culture

Adipose tissue was collected from human donors after cosmetic liposuction procedures. The collected tissue would have otherwise been discarded. The procurement of human adipose tissue was approved by the local bioethics committee (approval KB/85/A/2012). Human stromal vascular fraction (SVF) of adipose tissue was isolated using the method originally described by Zuk and coauthors [36]. The detailed procedure used for SVF isolation followed the one used in the former study [10]. The obtained SVF cells were seeded into T75 culture flasks at a density of 3 × 10^6^ nucleated cells per flask, and cultured at 37 °C and 5% CO_2_ in a humidified atmosphere. The complete culture medium (CM) consisted of Dulbecco’s Modified Eagle Medium (DMEM), 10% fetal bovine serum (FBS), and 1% antibiotic–antimycotic (all from Life Technologies, Carlsbad, CA, USA) supplemented with 5 ng/mL recombinant human fibroblast growth factor 2 (FGF-2) (Sigma Aldrich, St. Louis, MO, USA). Cells were cultured until reaching approximately 70% confluence, which usually occurred within 4–7 days. Then, the obtained ADSCs were cryopreserved in liquid nitrogen. Directly before each experiment, cells were thawed and further cultured in CM. The culture was passaged when 70–90% confluence was observed. The cells in Passage 2 or 3 were used in all experiments. Routinely performed isolation and culture techniques of ADSC lead to a population of cells wherein at least 95% of cells are positive for cell surface markers characteristic for mesenchymal stem cells: CD73, CD90, and CD105 and negative for hematopoietic (CD45) and endothelial (CD31) markers, as demonstrated by fluorescence-activated cell sorting (FACS) analysis in one of our previous studies [37]. The cells used for experiments were obtained from a laboratory, which holds a Good Manufacturing Practice (GMP) certificate and which produces the ADSC-based advanced therapy medicinal products (ATMP). ADSCs that are the active substance of the medicines produced in this laboratory were isolated and cultured in accordance with the approved procedures, under the pharmaceutical supervision. The quality and purity of cells meets criteria set by Mesenchymal and Tissue Stem Cell Committee of the International Society for Cellular Therapy which proposed minimal criteria to define human MSC.

### 2.2. Three-Dimensional Spheroid Culture

Three-dimensional ADSC spheroids were obtained as described in our former study [36]. Briefly, a low-attachment culture surface was prepared by coating 96-well round-bottom plates (Thermo Fisher Scientific Inc., Waltham, MA, USA) with a sterile-filtered, 10% *w/v* aqueous solution of Pluronic F127 (Sigma Aldrich). Afterward, 200 µl of a single-cell ADSC suspension containing 5 × 10^4^ cells/mL was added to each well. Consequently, 1 × 10^4^ cells were seeded for culture per well. The composition of the culture medium for each experiment is provided below. The prepared 96-well round-bottom plate was placed in a cell-culture incubator on a rotary shaker with gentle rotation. The 3D spheroids formed after approximately 24–48 h from seeding. The culture medium was replaced every 2–3 days.

### 2.3. Intracellular cAMP Level Regulation

To upregulate intracellular cAMP concentration, adenylate cyclase activator forskolin (FSK, purchased from Bio-Techne, Minneapolis, MN, USA) was added to the culture medium. In addition to forskolin, 500 µM of 3-isobutyl-1-methylxanthine (IBMX, purchased from Sigma Aldrich) was used to inhibit residual phosphodiesterases and downregulate cAMP degradation. To downregulate intracellular cAMP concentration, SQ 22,536 (Sigma Aldrich) was added to the culture medium. The exact final FSK and SQ 22,536 concentration in culture medium was 10 or 100 µM and depended on the type of the experiment, as described below. Medium containing cAMP regulators was replaced every 2–3 days as described by others [23].

### 2.4. Protein Kinase A Activity

ADSCs were seeded into 96-well flat-bottom plates at a density of 5 × 10^3^ cells per well for testing the 2D tissue culture polystyrene (TCPS) conditions or into 96-well, round-bottom, Pluronic-coated plates, as described above, for testing 3D spheroid conditions. Cells were seeded into a control medium (CM) deprived of FGF-2, or into osteogenic medium (OM) composed of DMEM with 10% fetal bovine serum (FBS) and 1% antibiotic–antimycotic (all from Life Technologies), supplemented with 10 nM dexamethasone, 3 mM NaH_2_PO_4_, and 50 µg/mL ascorbic acid 2-phosphate (all from Sigma Aldrich). Cells were maintained in OM and exposed to cAMP regulating conditions as described above.

Cultures were maintained for 48 h, and cells were then washed with PBS and lysed with a radioimmunoprecipitation assay buffer (RIPA buffer) (Thermo Fisher Scientific) with protease inhibitor cocktail cOmplete™ (Roche Diagnostics, Indianapolis, IN, USA); 50 µl of lysis buffer per well was used. Cells from two wells were pooled into one sample. Next, lysates were sonicated in a cold-water bath and briefly centrifuged to pellet the debris. PKA activity was assayed in each sample using a commercially available competitive ELISA–PKA Kinase Activity Kit (Enzo Life Science Inc., Farmingdale, NY, USA). For each assay reaction, 30 µl of the sample was added. The assay procedure was conducted according to the manufacturer’s instructions. Lastly, total protein concentration in the samples was determined using Pierce BCA assay (Thermo Fisher Scientific Inc.); the assay was performed according to the manufacturer’s instructions. Absorbance in the PKA assay and Pierce BCA assay was measured with FLUOstar plate reader (BMG Labtech GMBH, Ortenberg, Germany). PKA activity was expressed as the absorbance or as ng of active PKA normalized to the amount of protein used in each PKA reaction.

### 2.5. Intracellular cAMP Level Measurement

Intracellular cAMP level analysis was performed to select optimal cAMP regulation conditions. Experiments were done on cells isolated from a single donor to exclude donor-to-donor variability and facilitate statistically significant comparisons between treatment conditions.

First, ADSCs were detached from culture flasks to obtain a cell suspension in PBS. Cell concentration was adjusted to 1.33 × 10^6^ cells/mL, and 3.75 µl of the prepared suspension was added into each well of a 384-well plate. Next, an equal volume of cAMP-regulating compounds was added to achieve the desired final concentration of the tested compounds. Tested conditions were 10 nM SQ 22,536; 100 nM SQ 22,536; 10 nM FSK with 500 nM IBMX; and 100 nM FSK with 500 nM IBMX. For all treatment conditions, 10 nM dexamethasone was added to match with the osteogenic differentiation protocol, as this steroid is the OM component that was reported to upregulate cAMP level [38] by inhibiting cAMP phosphodiesterases [39]. Then, ADSCs were incubated at 37 °C for 1 or 4 h. Finally, the intracellular cAMP level was assayed with cAMP-Glo™ assay (Promega, Madison, WI, USA), using the procedure provided by the manufacturer. Luminescence was read using the FLUOstar plate reader (BMG Labtech). ADSCs treated with plain PBS were used as a reference for determining cAMP concentration. The cAMP level in the treated samples was expressed as a change in cAMP concentration with respect to the reference sample. The control sample, treated with PBS and 10 nM dexamethasone, was used for comparison with other treatment conditions.

### 2.6. Flow Cytometry—Cell Cycle

ADSCs were seeded into 6-well plates at a density of 1.5 × 10^5^ per well and cultured for 24 h in CM. Afterward, cAMP level regulators were added, 10 µM FSK and 500 nM IBMX or 100 µM SQ 22,536, and cells were further cultured for 24 h. ADSCs cultured in plain CM were used as the control. Then, cells were cultured with 5-ethynyl-2′-deoxyuridine (EdU) for 2 h to label the cells in the S phase of the cell cycle. Next, cells were detached from the culture surface with Accutase (Thermo Fisher Scientific). The collected cell suspension was fixed, and EdU was stained with Alexa Fluor 488 fluorochrome using reagents provided in the Flow Cytometry Cell Proliferation Assay Kit (Thermo Fisher Scientific) and according to the manufacturer’s instructions. Lastly, cells were stained with 7-amino-actinomycin D (7-AAD, Becton Dickinson, Franklin Lakes, NJ, US) to quantitatively label the DNA. Prepared samples were evaluated on a CytoFLEX flow cytometer (Beckman Coulter Inc., Indianapolis, IN, USA). Acquired data were analyzed using FlowJo software (FlowJo LCC, Ashland, USA). Cells in the G1/G0, S, and G2 phases of the cell cycle were gated on the basis of Alexa Fluor 488 and 7-AAD fluorescence. EdU-positive cells were considered S-phase cells, and 7-AAD-positive cells were assigned either to the G1/G0 or the G2 population depending on fluorescence intensity, which was increased in the G2 population by approximately twofold.

### 2.7. Flow Cytometry—Apoptosis

ADSCs were seeded into 6-well plates at a density of 1.5 × 10^5^ per well in CM and OM. After 24 h, cAMP level regulators were added to OM cultured cells; the applied concentration of regulators was the same as that for the cell-cycle experiments described above. Cells cultured in the medium containing 10% DMSO were included in the experiment as a positive control for apoptosis. Cells cultured in plain CM and OM were studied for comparison. ADSCs were cultured with cAMP level regulators and control media for 72 h. Afterward, the cells were stained using an Annexin V Apoptosis Detection Kit (Becton Dickinson) according to the manufacturer’s instructions. Briefly, cells were detached from culture plates with Accutase (Thermo Fisher Scientific), washed in PBS, and resuspended in the binding buffer provided in the kit. Next, Annexin V conjugated with fluorescein isothiocyanate (FITC) and 7-AAD solutions were added, and cells were incubated for 15 min at room temperature. After incubation, samples were immediately analyzed on a CytoFLEX flow cytometer (Beckman Coulter Inc.), and the acquired data were analyzed with FlowJo software (FlowJoLCC). Annexin V-positive cells were gated as early apoptotic, 7-AAD-positive cells were gated as necrotic/nonviable cells, and Annexin V and 7-AAD double-positive cells were gated as apoptotic/nonviable cells.

### 2.8. Early and Late Osteogenic Differentiation

The influence of cAMP level on the osteogenic differentiation of ADSCs was studied using two different experiment protocols. In the first protocol, which was used to study early osteogenic differentiation, ADSCs were seeded into 6-well plates for studying 2D TCPS conditions, or into 96-well, round-bottom, Pluronic-coated plates for studying 3D spheroid conditions. Cells were cultured in OM or in OM supplemented with cAMP level regulators. To upregulate cAMP level, 10 µM FSK and 500 nM IBMX were used, while 100 µM SQ 22,536 was used to downregulate cAMP level. After 2 or 7 days of culture, cells were lysed for RNA extraction and the analysis of *RUNX2*, *SP7* (Osterix), and *IBSP* gene expression, as described below.

In the second protocol, used for studying late osteogenic differentiation, ADSCs were seeded into 96-well, round-bottom, Pluronic-coated plates to generate the 3D spheroids. Then, the ADSC spheroids were then cultured for 7 days in OM to obtain osteogenically predifferentiated ADSCs. Then, the spheroids were disassociated by treatment with Accumax (Thermo Fisher Scientific). The obtained single-cell suspension was seeded into standard 24-well culture plates (2D TCPS conditions) at a density of 3 × 10^4^ cells per well. The newly seeded cells were cultured in OM with 10 nM 1α, 25-dihydroxyvitamin D3 (OM-D3). Optionally, cAMP level regulators were added to the OM-D3 medium, as described above for the early osteogenic differentiation experiments. Next, ADSCs were cultured for an additional period of 7 or 14 days; therefore, the whole experiment, including the predifferentiation stage, lasted 14 or 21 days. After a total of 14 or 21 days, cells were fixed for bone-mineral and osteocalcin staining or lysed for Western blot analysis as described in the respective sections below. A schematic diagram summarizing the experiment conditions used for studying ADSC osteogenic differentiation is shown in Figure 5.

### 2.9. Gene-Expression Analysis

Cells were lysed directly into culture dishes with a Fenzol buffer (A&A Biotechnology, Gdynia, Poland), and lysates were immediately frozen in −80 °C for storage. After thawing, total RNA was isolated with Total RNA Mini Plus Concentrator kit (A&A Biotechnology) using the provided microcolumns. Procedures were carried out according to the manufacturer’s instructions. The concentration and purity of RNA were determined with a NanoDrop Spectrophotometer (Thermo Fisher Scientific Inc., Waltham, MA, USA). Next, samples were treated with RNase-free DNase to remove possible contamination of genomic DNA (A&A Biotechnology). The purified RNA was reverse-transcribed with a High Capacity cDNA Synthesis Kit (Thermo Fisher Scientific Inc.) on a 7500 Fast thermal cycler (Applied Biosystems, Carlsbad, CA, USA). Each reverse-transcription reaction contained 600 ng of total RNA. Equal amounts of the prepared cDNA were used in Real-Time PCR reaction, which was carried out using TaqMan^®^ Universal PCR Master Mix (Thermo Fisher Scientific Inc.) on a 7500 Fast Thermal Cycler. The following TaqMan assays (Thermo Fisher Scientific Inc.) were used in PCR reactions to amplify the target genes: *runt-related transcription factor 2* (*RUNX2*, Hs00231692_m1), *Osterix* (*SP7*, Hs01866874_s1), *bone sialoprotein* (*IBSP*, Hs00173720_m1), *inhibitor of DNA binding 2* (*ID2*, Hs04187239_m1) and *ubiquitin C* (*UBC*, Hs00824723_m1). In a single experiment, each transcript was assayed in triplicate. Relative gene expression was determined using the comparative ΔΔCt method, using UBC as the reference gene and undifferentiated ADSCs as the reference sample. Gene expression analysis experiments were performed on cells obtained from three donors.

### 2.10. Bone-Mineral Staining and Quantification

At Day 14 or 21 of culture, cells cultured in 24-well plates were fixed in 10% buffered formalin (Sigma Aldrich). The accumulated bone mineral was fluorescently stained with OsteoImage kit (Lonza Ltd., Basel, Switzerland) according to the manufacturer’s instructions. Cell nuclei were counterstained with Hoechst 33,342 (Sigma Aldrich). Undifferentiated ADSCs were stained following the same procedure to validate bone mineral staining specificity. The bone mineral was semiquantified by measuring fluorescence intensity at 488/520 nm, while the relative number of cells was semiquantified by measuring Hoechst 33,342 florescence intensity at 355/460 nm (excitation/emission). Fluorescence intensity was measured in 10 points evenly distributed within a well. The relative quantity of bone mineral was expressed as OsteoImage fluorescence intensity normalized to Hoechst 33,342 fluorescence intensity. Each sample was assayed in quadruplicate. Additionally, stained samples were observed under a Nikon Eclipse-Ti inverted microscope (Nikon Instruments Inc., Melville, NY, USA) for image acquisition.

### 2.11. Immunofluorescence Staining

Cells were fixed as described above at Day 14 or 21. Afterward, cells were incubated in 10% normal goat serum in PBS solution to block nonspecific antibody binding. Cell membranes were permeabilized by incubation with a 0.1 Triton-X solution in PBS. Next, cells were incubated with a solution of a mouse monoclonal anti-osteocalcin antibody (R&D Systems Inc., Minneapolis, MN, USA) at 4 °C overnight. Excess antibody was washed, and samples were stained with a goat anti-mouse Cy5-conjugated antibody (Thermo Fisher Scientific Inc.). Lastly, samples were washed, and nuclei were counterstained with Hoechst 33342. Undifferentiated ADSCs were processed to provide control of osteocalcin-staining specificity. Samples were observed under a Nikon Eclipse-Ti inverted microscope (Nikon Instruments Inc.).

### 2.12. Western Blot

ADSCs at Day 14 or 21 were directly lysed with RIPA buffer (Thermo Fisher Scientific Inc.) in 6-well culture dishes. Pierce BCA assay (Thermo Fisher Scientific Inc.) was performed to determine total protein concentration in lysates; the BCA reaction was carried out according to conditions specified by the kit manufacturer. Absorbance measurement after the reaction was performed with a FluorStar plate reader (BMG Labtech). Next, lysates were mixed with Laemmli sample buffer containing 10% *v/v* beta-mercapto-ethanol and incubated at 100 °C for 5 min. Samples containing 10 µg of total protein were loaded on 10% sodium dodecyl sulfate-polyacrylamide gel (National Diagnostics, Atlanta, USA). After running electrophoresis, the protein was blotted onto an immobilon-P membrane (Merck Millipore, Billerica, MA, USA) using a semidry blotting apparatus (Thermo Fisher Scientific). After blocking, the membrane was incubated with a solution of mouse monoclonal anti-osteocalcin antibody (Thermo Fisher Scientific Inc.) or mouse monoclonal anti-β-actin antibody (Merck Millipore). Target protein bands were detected by incubating the membrane with anti-mouse antibody conjugated with horseradish peroxidase (both Thermo Fisher Scientific), and eliciting a chemiluminescence reaction with enhanced chemiluminescent (ECL) horseradish peroxidase substrate (Thermo Fisher Scientific). Bands were visualized on photographic film (Sigma Aldrich). Densitometric analysis of blot images was performed with ImageJ software (Schneider et al., 2012) to obtain semiquantitative osteocalcin levels. Experiments were performed on cells isolated from three donors.

### 2.13. Statistical Analysis

All experiments were performed at least three times on cells isolated from different donors unless indicated otherwise. In total, cells obtained from 6 donors were used for all experiments.

Quantitative data from all experiments were analyzed with STATISTICA software (StatSoft Inc., Tulsa, OK, USA), while graphs were prepared using Microsoft Excel. Presented values represent the mean from all three experiments or the mean from the replicates for each experiment separately. Error bars in the graphs indicate standard deviation or ±95% confidence interval of the mean, as indicated in the figure legends. ANOVA with post hoc Tukey test was used to assess the statistical significance between experiment groups. The difference between experiment groups was considered statistically significant when the calculated *p*-value was 0.05 or lower.

## 3. Results

### 3.1. PKA Activity in 2D and 3D Osteogenic Culture

**Figure 1:**
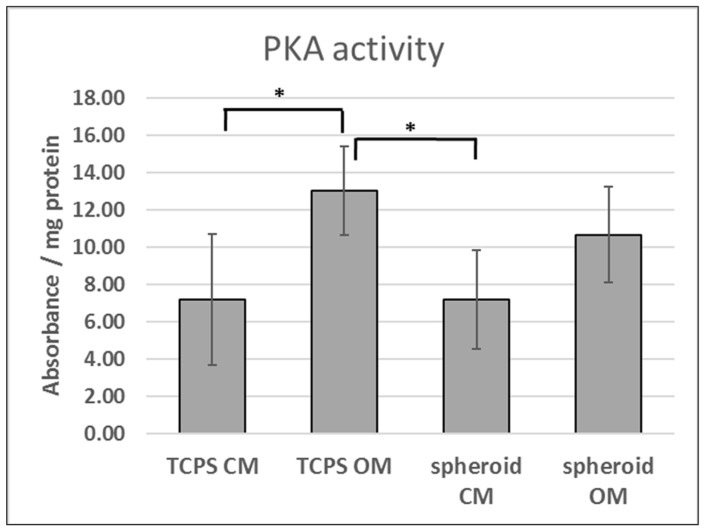
Activity of protein kinase A (PKA) in standard 2D culture and 3D spheroids. TCPS, tissue culture polystyrene (standard 2D conditions); CM, culture medium; OM, osteogenic medium. Means shown from three experiments performed on cells obtained from three donors (*n* = 9). Error bars, standard deviation. *, *p* < 0.05.

We speculated that the improved osteogenic commitment of ADSCs in spheroids might be attributed to altered cAMP signaling. Therefore, PKA activity was analyzed in ADSCs cultured in standard 2D culture dishes (TCPS) or in the form of 3D spheroids and maintained in culture medium (CM) or osteogenic medium (OM) for 2 days (Figure 1). PKA activity in ADSCs cultured in OM was elevated compared to CM conditions on 2D TCPS. However, no statistically significant differences in PKA activity were observed between OM and CM conditions when ADSCs were cultured in 3D spheroids.

### 3.2. Effect of Adenylate Cyclase Regulation on cAMP Pathway Activation

**Figure 2:**
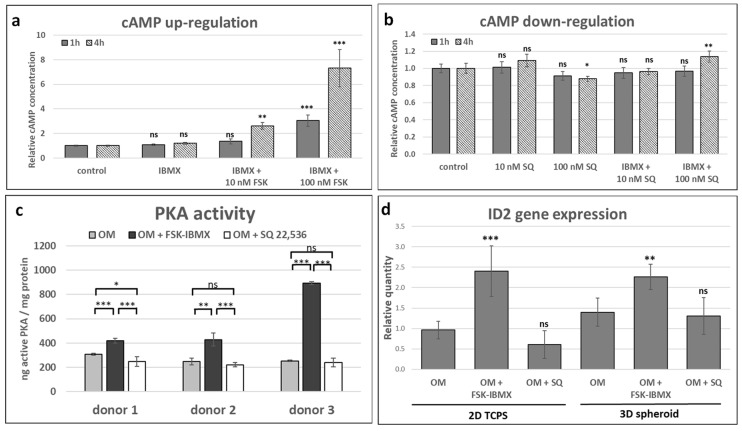
Effect of adenylate cyclase regulation on cAMP pathway activity. Cells cultured in OM and treated with adenylate cyclase activator forskolin (FSK, concentration of 10 nM unless indicated otherwise) and phosphodiesterase inhibitor 3-isobutyl-1-methylxanthine (IBMX, 500 nM), or with adenylate cyclase inhibitor SQ 22,536 (100 nM unless indicated otherwise). Analysis of intracellular cAMP under cAMP (**a**) upregulation and (**b**) downregulation. Cells in suspension treated with regulator for 1 or 4 h, and each treated sample contained 5000 cells. Relative cAMP concentration, normalized to control sample, is shown. (**a**,**b**) Mean from three experiments performed on cells isolated from a single donor, error bars represent standard deviation. (**c**) protein kinase A (PKA) activity assay. Cells cultured with regulators for 48 h. PKA activity shown as ng of active PKA enzyme, normalized to the amount of total protein in sample. Mean from three experiments performed on cells isolated from different donors; results from each cell donor separately presented, error bars represent standard deviation. (**d**) Relative expression of *ID2* gene, cyclic–AMP response element binding protein (CREB) target gene. Cells cultured with regulators for 48 h. Mean from three experiments performed on cells isolated from different donors. Control, undifferentiated cells; “0”, cells cultured in OM without regulators. Error bars, ±95% confidence interval. Statistical significance between control or OM and adenylate cyclase regulation conditions is marked: *, *p* < 0.05; **, *p* < 0.01; ***, *p* < 0.001; ns, not significant.

Next, we aimed to select the optimal conditions of adenylate cyclase regulation in ADSCs, which would provide the stimulation or inhibition of the signaling pathway. Therefore, intracellular cAMP concentration was assayed in ADSC suspension obtained from 2D TCPS culture (Figure 2a,b). Cells in suspension were treated with only 3-isobutyl-1-methylxanthine (IBMX), FSK and IMBX, SQ 22,536, or SQ 22,536 and IBMX. We initially selected the 10 and 100 nM concentrations of FSK and 500 nM IBMX for testing on the basis of results of previously published studies [15,24,40]. Treatment of ADSCs with only IBMX did not increase cAMP level, but both 10 and 100 nM FSK combined with IBMX for 4 h resulted in the significant upregulation of cAMP concentration. In contrast, treatment with 10 nM SQ 22,536 did not downregulate cAMP concentration. However, the use of 100 nM SQ 22,536 did result in a significant decrease in cAMP concentration after 4 h treatment. Therefore, 10 nM FSK combined with IBMX and 100 nM SQ 22,536 were selected for further experiments, as these conditions were found to effectively regulate adenylate cyclase activity.

Next, we aimed to study the downstream points of the cAMP signaling cascade upon treatment with FSK and SQ 22,536. Protein kinase A (PKA) activity assay was performed on ADSCs cultured in 2D TCPS for 2 days in OM with cAMP regulators (Figure 2b). PKA activity in ADSCs obtained from three donors was enhanced in elevated cAMP conditions, i.e., upon treatment with FSK and IBMX. On the other hand, PKA activity was inhibited in downregulated cAMP conditions, i.e., upon treatment with SQ 22,536, in ADSC populations obtained from one out of three donors. PKA activity in the two remaining ADSC populations did not change in response to SQ 22,536.

Further, we assessed the expression of the *ID2* gene (Figure 2c), which is a known cAMP/PKA/CREB pathway target in BMSC [22]. As expected, elevated cAMP conditions resulted in the upregulation of *ID2* gene expression both in the 2D TCPS and the 3D spheroids after 2 days of culture in OM. However, cAMP downregulation did not inhibit *ID2* gene expression in the tested conditions.

Stimulation of the cAMP pathway by FSK and IBMX was confirmed at all of the three tested signaling levels. However, SQ 22,536 only downregulated the signaling pathway at the level of adenylate cyclase activity. The lack of regulation of PKA activity and *ID2* gene expression by SQ 22,536 might have been caused by the action of additional regulatory mechanisms that compensated for the effect of low cAMP in the proposed experiment conditions.

### 3.3. Cell-Cycle Progression

**Figure 3:**
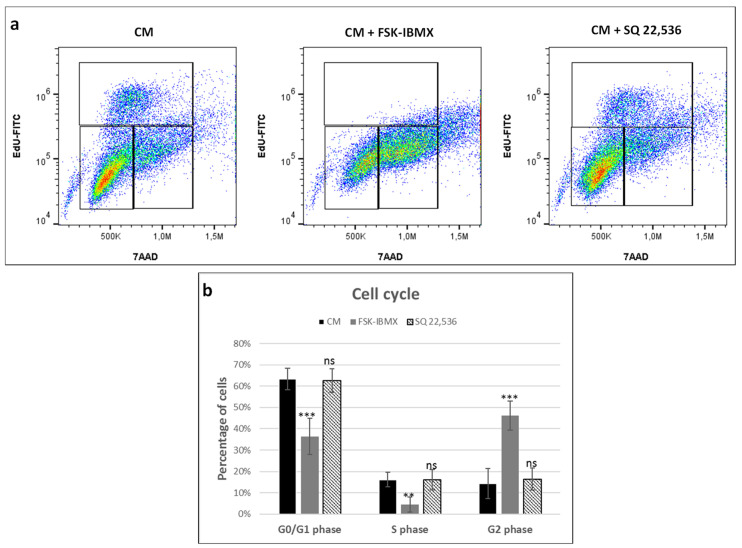
Effect of cAMP pathway regulation on cell cycle. Cells cultured in CM and treated with adenylate cyclase activator forskolin (FSK, 10 nM) and phosphodiesterase inhibitor 3-isobutyl-1-methylxanthine (IBMX, 500 nM), or with adenylate cyclase inhibitor SQ 22,536 (100 nM) for 24 h and pulsed with 5-ethynyl-2′-deoxyuridine (EdU). After treatment, cells were detached from culture dishes and labeled with azide EdU-AlexaFluor 488 and 7-amino-actinomycin D (7-AAD) and analyzed on a flow cytometer. (**a**) Representative dot plots from flow cytometry analysis. (**b**) Mean G0/G1-, S-, and G2-phase population counts from experiments performed on cells isolated from three donors; error bars represent standard deviation. **, *p* < 0.01; ***, *p* < 0.001; ns, not significant.

We further aimed to assess the effect of cAMP regulators on ADSC proliferation by analyzing the cell-cycle phases in EdU-pulsed and 7-AAD-labeled cells (Figure 3). ADSC were cultured in 2D TCPS in CM with cAMP regulators for 24 h before flow cytometry analysis. We choose CM and not OM in order to avoid the potential influence of pro-osteogenic additives on cell cycle. Treatment with FSK–IBMX downregulated the percentage of S-phase cells (EdU-positive cells) from 16.0% to 4.5%. At the same time, G2-phase cells’ population increased in FSK–IBMX-treated cells. Therefore, obtained results suggested cell-cycle arrest in the G2 phase under cAMP-elevated conditions. On the other hand, treatment with SQ 22,536 did not have a significant effect on the cell cycle.

### 3.4. Apoptosis Induction

**Figure 4:**
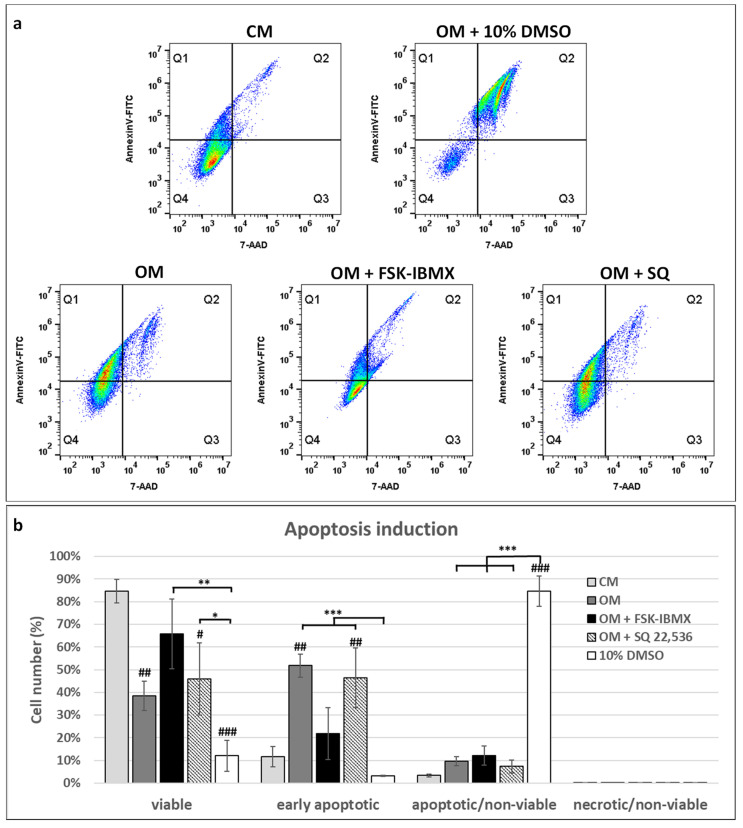
Effect of cAMP pathway regulation on apoptosis. Cells cultured in OM and treated with adenylate cyclase activator forskolin (FSK, 10 nM) and phosphodiesterase inhibitor 3-isobutyl-1-methylxanthine (IBMX, 500 nM) or with adenylate cyclase inhibitor SQ 22,536 (100 nM) for 72 h. Cells cultured in CM and OM with 10% DMSO were used as a reference for low and high apoptosis, respectively. Prior to flow-cytometry analysis, cells were labeled with Annexin V-FITC and 7-AAD. (**a**) Representative dot plots from flow cytometry analysis. (**b**) Mean population counts from three experiments performed on cells isolated from different donors; error bars represent standard deviation. Viable cells: Annexin V-/7-AAD–, early apoptotic cells: Annexin V+/7-AAD–, apoptotic/nonviable cells: Annexin V+/7-AAD+, necrotic/nonviable cells Annexin V-/7-AAD+. *, *p* < 0.05; **, *p* < 0.01; ***, *p* < 0.001; ns, not significant; #, statistical significance of population count between marked experiment condition and CM sample (reference sample); number of # symbols denotes *p*-value as described above.

The observed cell-cycle inhibition in FSK–IBMX-treated cells prompted us to study apoptosis induction (Figure 4). ADSC were treated with FSK–IBMX or with SQ 22,536 in OM for 3 days; then, cells were detached from culture dishes for combined Annexin V and 7-AAD staining.

Early apoptotic cells were detected on the basis of positive Annexin V and negative 7-AAD staining, while nonviable cells were detected by their positive 7-AAD staining. ADSC populations cultured in CM contained only 11.7% and 3.4% of early apoptotic and nonviable cells, respectively. Interestingly, switching ADSCs from the CM to the OM caused a significant increase in the percentage of apoptotic cells (Annexin V-positive cells). In OM, 51.8% of cells were early-apoptotic, i.e., stained positive with Annexin V and negative with 7-AAD. On the other hand, FSK–IBMX treatment significantly decreased the population of early-apoptotic cells to 21.9% of all cells cultured in OM; at the same time, SQ 22,536 did not have an effect on the number of early-apoptotic cells in OM. These results indicate that inhibition of the cell cycle observed in FSK–IBMX-treated cells did not originate from the induction of cell death. In fact, FSK–IBMX treatment protected ADSC from apoptosis induction caused by the OM.

### 3.5. Osteogenic Differentiation

**Figure 5:**
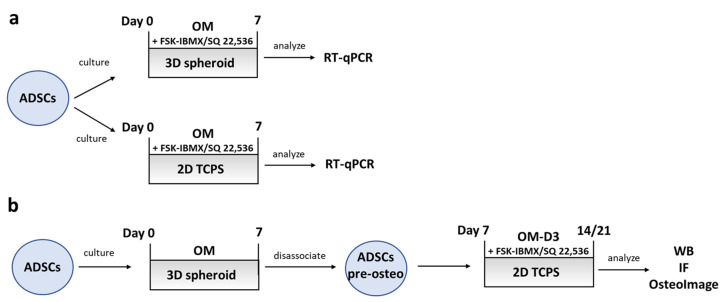
Early- and late-osteogenesis experiment procedures. Experiment setup for studying early and late osteogenesis of adipose-derived mesenchymal stem cells (ADSCs). (**a**) Early and (**b**) late osteogenesis.

A schematic diagram summarizing the experiment conditions for studying the effect of cAMP regulation on early and late ADSC osteogenesis is shown in Figure 5.

#### 3.5.1. Early Osteogenic Differentiation

**Figure 6:**
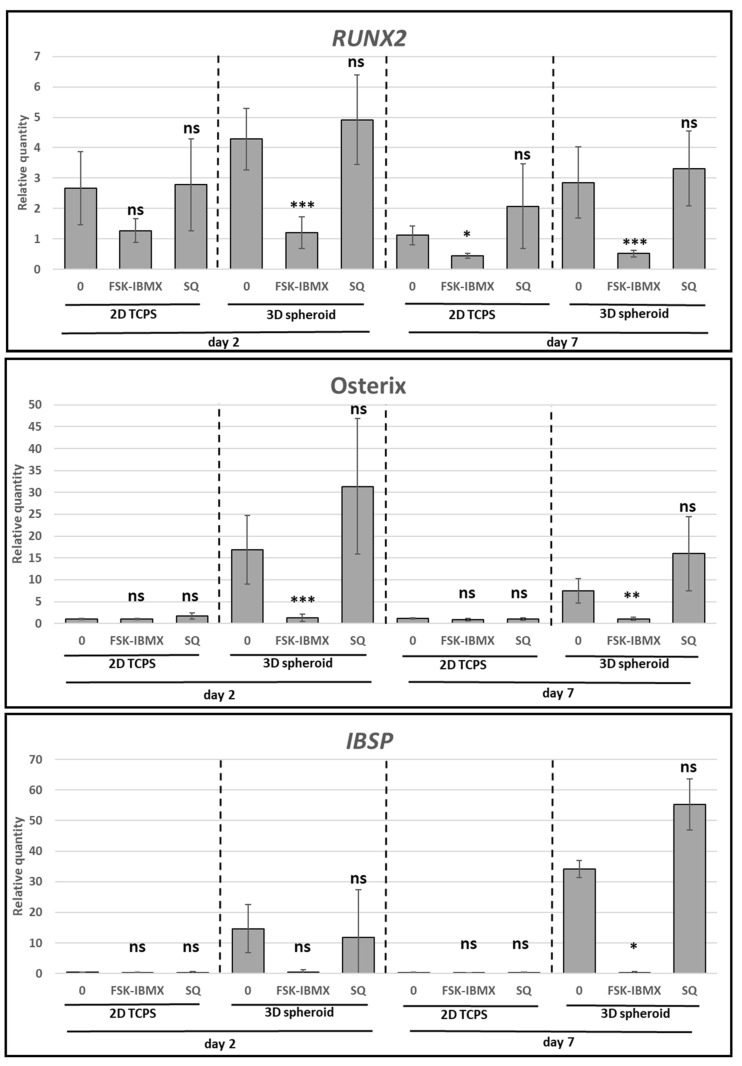
Effect of cAMP pathway regulation on osteogenesis—expression of preosteoblast characteristic genes. ADSCs differentiated for 7 days on 2D TCPS or in 3D spheroids in osteogenic medium (0), optionally containing FSK–IBMX (FSK, 10 nM; IBMX, 500 nM) or SQ 22,536 (SQ, 100nM). Gene-expression analysis performed on Days 2 and 7. Presented data are mean from three experiments; each experiment was performed on cells isolated from a different donor. Error bars represent ±95% confidence interval. Statistical significance between “0” sample and FSK–IBMX and SQ at the given time point and culture method marked with asterisks: *, *p* < 0.05; **, *p* < 0.01; ***, *p* < 0.001; ns, not significant.

Next, the effect of cAMP level changes on early and intermediate events of ADSC osteogenic differentiation was analyzed (Figure 6). The expression of *RUNX2, Osterix, and IBSP* genes was analyzed in 2D TCPS and 3D spheroid conditions at Days 2 and 7 of treatment. Surprisingly, the upregulation of the cAMP level caused the downregulation of *RUNX2* expression in both 2D and 3D conditions. However, the downregulation of cAMP level had no significant effect on *RUNX2* expression. Furthermore, *Osterix* and *IBSP* gene expression were downregulated in high cAMP conditions to the level of undifferentiated control cells. However, the effect of high cAMP was only observed in 3D spheroid cultures, where the OM significantly upregulated *Osterix* and *IBSP* gene expression compared to in undifferentiated cells. Changes in gene expression occurred either at Day 2 or 7 of the culture, depending on culture dimensionality and used donor cells. On the other hand, the expression of *Osterix* and *IBSP* genes was not significantly changed in low cAMP conditions.

The provided data showed that the commitment of ADSCs to the osteoblast lineage was inhibited under a high concentration of intracellular cAMP and high PKA activity.

#### 3.5.2. Late Osteogenic Differentiation

**Figure 7:**
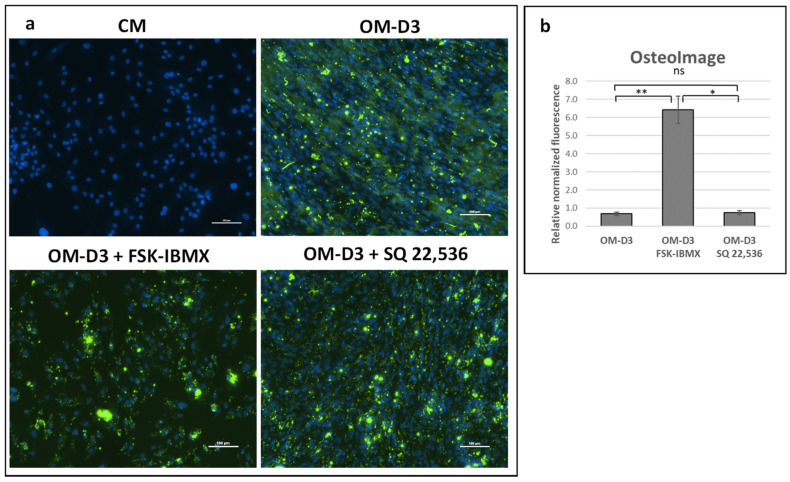
Effect of cAMP on late differentiation events after predifferentiation in spheroids—bone-mineral production. ADSCs predifferentiated in spheroids for 7 days in OM and differentiated for another 14 days on 2D TCPS (total differentiation time was 21 days) in osteogenic-maturation medium (OM-D3) with FSK–IBMX (FSK, 10 nM; IBMX, 500 nM) or SQ 22,536 (100 nM). (**a**) Bone mineral stained with OsteoImage kit (Lonza) and observed under fluorescence microscope (green). Nuclei stained with Hoechst 33,342 (blue). (**b**) Green and blue fluorescence intensity measured with plate reader to semiquantify the bone-mineral amount relative to number of cells in culture. Mean from three experiments performed on cells isolated from different donors is shown, error bars represent standard deviation. *, *p* < 0.05; **, *p* < 0.01; ns, not significant.

**Figure 8:**
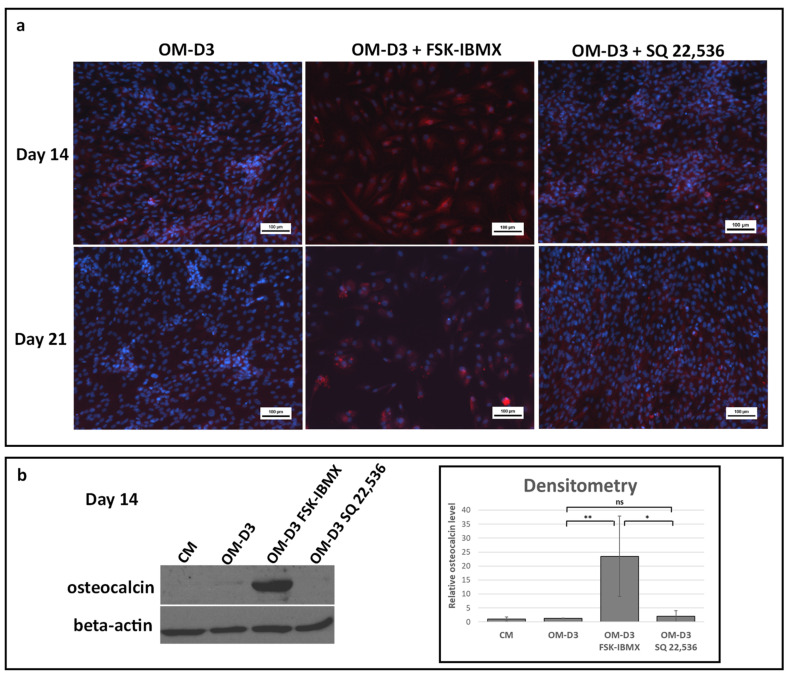
Effect of cAMP on late differentiation events after predifferentiation in spheroids—protein production. ADSCs predifferentiated in spheroids for 7 days in OM and differentiated for another 14 days on 2D TCPS (total differentiation time was 21 days) in OM-D3 with FSK–IBMX (FSK, 10 nM; IBMX, 500 nM) or SQ 22,536 (100 nM). (**a**) Osteocalcin protein stained with mouse monoclonal antibody and detected with goat anti-mouse Alexa Fluor 700 secondary antibody (red). Nuclei stained with Hoechst 33,342 (blue). (**b**) Western blot for osteocalcin and beta-actin performed at Day 14. Representative blot and densitometric analysis of osteocalcin level shown. Osteocalcin level was normalized to beta-actin. Mean from three independent experiments is shown; each experiment was performed on cells isolated from a different donor. Error bars represent standard deviation. *, *p* < 0.05; **, *p* < 0.01; ns, not significant.

For the analysis of the late stages of osteogenic differentiation, cells were first predifferentiated in OM in 3D spheroids for 7 days. Next, cells were transferred into a 2D TCPS culture and further maintained up to Day 14 or 21 in the osteogenic-maturation medium (OM-D3). At the osteogenic-maturation stage, i.e., starting at Day 7 of the experiment, cAMP level regulation with FSK and IBMX or SQ 22,536 was performed, with plain OM-D3 serving as a reference culture. Bone-mineral (Figure 7) and osteocalcin-protein production (Figure 8) were analyzed.

Mineral production at Day 14 was not observed in any of the maturation conditions (data not shown). However, on Day 21, the mineral was present in all analyzed conditions. Quantitative analysis of fluorescence intensity indicated that the amount of bone mineral relative to cell number was over sixfold higher in FSK–IBMX-treated cultures compared to that in the reference and SQ 22,536 cultures (Figure 7). Treatment with SQ 22,536 had no effect on the amount of produced bone mineral.

Next, the production of osteocalcin, a protein characteristic to mature osteoblasts, was analyzed with immunofluorescence and Western blot. A low level of osteocalcin was detected with immunofluorescence staining both at Days 14 and 21 in OM-D3. Osteocalcin staining was more intensive in FSK–IBMX-treated cultures, while treatment with SQ 22,536 had no effect on osteocalcin staining (Figure 8a). The qualitative observations were confirmed by semiquantitative Western blot analysis of samples collected at Day 14 (Figure 8b). Osteocalcin level in ADSCs treated with FSK–IBMX was significantly higher than that in the OM-D3 and OM-D3 SQ 22,536 conditions.

Bone-mineral staining and osteocalcin-protein analysis indicated that cAMP upregulation in osteogenically predifferentiated ADSCs enhances late differentiation events.

## 4. Discussion

In this study, we analyzed the influence of intracellular cAMP level on ADSC behavior in in vitro culture, with an emphasis on its role in ADSC osteogenic differentiation.

Former studies performed at our laboratory [35] and by others [31,41] showed that the differentiation of MSCs to osteoblasts can be enhanced by the application of a 3D spheroid culture. Therefore, it was intriguing whether cAMP pathway activity would differ upon the osteogenic induction of ADSCs cultured in standard 2D monolayer and 3D spheroid culture. PKA activity was significantly upregulated in 2D TCPS culture in the osteogenic medium compared to the control culture medium. The upregulation of PKA activity in OM could be attributed to the presence of dexamethasone, which was found to inhibit cAMP phosphodiesterase 4, which is a cAMP-hydrolyzing enzyme [39]. On the other hand, there was no statistically significant difference in PKA activity between control-culture medium and osteogenic medium in 3D spheroid conditions. This result suggested that PKA activity might play a role in spheroid-induced osteogenic commitment; the inhibition of PKA upregulation after exposure to the osteogenic medium may provide improved osteogenic commitment. In a study on human BMSCs, inhibition of IBMX- or forskolin-induced cAMP pathway activity resulted in inhibited PPARG and upregulated *RUNX2* expression, indicating that the limitation of cAMP activity might direct MSCs into an osteogenic lineage [24]. Nonetheless, further studies are needed to establish how PKA signaling is affected by the spheroid formation process.

However, we cannot correlate differences in PKA activity observed in 2D TCPS versus 3D spheroids with cAMP level due to a prolonged time of spheroid formation (24–48 h) and the fact that changes in cAMP level can be observed only for several hours after stimulation.

The upregulation of cAMP level in ADSCs was performed with a small-molecule compound, a well-established adenylate cyclase agonist, forskolin, which activates a broad spectrum of adenylate cyclase isoforms and demonstrated efficacy toward many cell and tissue types [42]. SQ 22,536, an inhibitor of adenylate cyclase, is one of the most widely applied in cell-culture studies [43], and it was used in this study with the aim of downregulating cAMP level. Although both compounds have been extensively used in cell-culture applications, ADSCs were not among tested cell types. Therefore, the effect of regulators on intracellular cAMP level was verified, and an optimal concentration was chosen. As expected, forskolin upregulated and SQ 22,536 downregulated cAMP level in ADSCs. In addition, an IBMX-phosphodiesterase inhibitor was selected alongside FSK for cAMP level upregulation. Afterward, the effect of cAMP level regulation on downstream-signaling elements was assessed. PKA activity and *ID2* gene expression were upregulated in ADSCs treated with FSK and IBMX, which suggested that the cAMP molecular signal was transduced through PKA and possibly by CREB, although other ID2 regulators could be involved, such as β-catenin [44] and BMP-6 [45]. However, other PKA targets, as well as the cAMP-regulated protein (EPAC) [46], may also be activated following cAMP upregulation. On the other hand, cAMP downregulation by SQ 22,536 did not result in significant PKA activity downregulation in two out of the three donors, while no significant change in *ID2* gene expression was observed in ADSCs originating from all three tested donors. This might have been caused by insufficient or transient cAMP level downregulation. Additionally, the basal level of PKA activity might have already been low prior to treatment, so the expected effect on enzyme activity might not have been observed after downregulation of cAMP concentration with SQ 22,536. After the selection of the optimal protocol for cAMP regulation in ADSCs, further studies were performed to elucidate the role of cAMP concentration on ADSC performance.

It is well established that cAMP signaling may regulate MAP kinase activity and consequently regulate the proliferation of cells [47]. cAMP activity may have both stimulatory and inhibitory effects on ERK kinase, which directly regulates cell proliferation [47].

Therefore, cell-cycle progression was studied in ADSCs cultured in 2D TCPS upon the regulation of cAMP level. Cells cultured in the control medium proliferated efficiently, with 16% of cells in the analyzed population in the S phase of the cell cycle. The downregulation of cAMP level did not have any effect on the percentage of cells in the S phase. However, the upregulation of cAMP level significantly decreased the number of cells in the S phase to 4.5% following treatment. At the same time, the percentage of cells in the G2 phase significantly increased upon cAMP upregulation and reached 46.1%, compared to approximately 15% in control medium and in cAMP downregulation conditions. Therefore, the upregulation of cAMP level decreased the proliferation rate of ADSCs. The increase of G2-phase cell populations suggests that proliferation inhibition might have occurred due to an arrest in the G2 phase. However, results of other studies on the effect of cAMP upregulation in MSCs have produced ambiguous results. Treatment of umbilical-cord-blood-derived MSCs with prostaglandin E2 resulted in the upregulation of cAMP signaling and at the same time increased the cell-proliferation rate [48]. On the other hand, the treatment of skin-derived MSCs with a cAMP analog, db-cAMP, inhibited the proliferation of cells in the serum-containing medium [49]. Similarly, the 8-CPT-cAMP analog inhibited the proliferation of human ADSCs, which agrees with our results. Therefore, the effect of cAMP on the proliferation of MSCs seems to be dependent on the origin of the cells. Presumably, MSC types, of which the proliferation depends on ERK signaling, may be negatively regulated by cAMP signaling, although this hypothesis needs experimental verification.

Due to the cell-cycle inhibitory effect of elevated cAMP in ADSCs, we also investigated the role of cAMP in ADSC apoptosis. Interestingly, OM caused a significant upregulation of apoptosis in ADSCs, but OM-induced apoptosis was alleviated by FSK–IBMX treatment. Therefore, we showed that elevated cAMP actually protects ADSCs from apoptosis.

Literature data showed that cAMP can act both as a pro- and antiapoptotic agent, depending on the cell type under investigation. For example, increased apoptosis under high cAMP concentration was observed in cardiomyocytes [50] and lymphoma cells [51], while neurons [52], gastric epithelial cells [53], and hepatocytes [54] were protected from apoptosis by cAMP signaling induction. This dual effect can be explained by two downstream effector proteins induced by cAMP, PKA or EPAC, which promote or inhibit apoptosis, respectively [55]. When it comes to MSCs, it was reported that rat bone-marrow MSCs were protected from glucose and oxidative-stress-induced apoptosis by the exendin-4 protein acting through cAMP signaling [56]. On the other hand, 8-CPT–cAMP upregulated irradiation-induced apoptosis in human ADSCs by inhibiting cyclin E degradation [57]. Therefore, the biological effect of cAMP is dependent on the cellular context—in this case, on cell type and possibly also on the type of apoptosis-inducing factor. Our study contributes to the wealth of research on apoptosis-regulating stimuli in mammalian cells, providing more insight into the differentiation-induced apoptosis of ADSCs.

Furthermore, early and late osteogenic differentiation was analyzed in ADSCs under cAMP upregulation and downregulation. Early osteogenesis was significantly inhibited under high intracellular cAMP concentration. On the other hand, late osteogenesis was significantly stimulated after treatment with cAMP-elevating agents. Therefore, the effect of cAMP signaling on ADSC osteogenesis seems to be dependent on the cellular context, i.e., on the osteogenic-maturation stage of cells that are subjected to cAMP concentration regulation. Additionally, the 3D spheroid culture was found to provide a valuable experiment setup for the study of ADSC osteogenesis regulation, as it provides an efficient induction of osteogenesis in a growth-factor-free, standard differentiation medium.

Results already published in the literature seem to support this hypothesis. The treatment of human bone marrow MSCs with forskolin or db-cAMP upregulated both early and late osteogenesis, i.e., it increased ALP activity and upregulated BMP-2 secretion and mineralization [15]. The pretreatment of bone-marrow MSCs with db-cAMP enhanced mineralization on a ceramic scaffold in the mouse ectopic osteogenesis model [22]. Another activator of the cAMP/PKA/CREB signaling pathway, CW008, was reported to in vitro enhance ALP activity and mineralization in bone-marrow MSCs [21]. Interestingly, the same study reported that the induction of PKA signaling inhibits the secretion of leptin [21], which is a hormone produced by adipocytes. Results from studies utilizing chemical treatment to manipulate the level of cAMP were confirmed in the Gsα knock-out mouse model. In vivo, the knock-out of Gsα in *Osterix*-expressing preosteoblasts caused marrow adiposity and osteoporosis. Similarly, MSCs with dysfunctional Gsα showed an increased expression of *PPARG, CABPA, and FABP4*, which are adipogenesis-characteristic genes [58]. These results confirmed the importance of cAMP signaling in the osteogenic differentiation of bone-marrow MSCs. Lastly, environmental stimuli, namely, oscillatory fluid shear force, was found to induce osteogenic differentiation via the cAMP pathway, as was reported in a recent study that utilized murine MSC cell line C3H10T1/2 [59].

Contrary to other studies, a report on bone-marrow MSC osteogenesis showed that treatment with IBMX and forskolin enhanced adipogenesis, while the inhibition of PKA enhanced early osteogenesis [24]. Interestingly, this study also reported the inhibition of leptin secretion under increased cAMP signaling [24]; nonetheless, the biological outcome was different than the one reported by Kim et al. [21]: the induction of adipogenesis at the expense of osteogenesis. Additionally, prolonged exposure of rat bone-marrow MSCs to forskolin decreased mineralization in vitro [23], suggesting that long-term cAMP signaling induction may actually downregulate osteoblast maturation.

Studies on ADSCs add even more ambiguity to the role of cAMP on MSC osteogenesis. For example, it was reported that the adipogenic differentiation of human ADSCs is stimulated by both 8-CTP-cAMP and IBMX. The activation of adipogenesis was mediated through the PKA- and EPAC-dependent signaling pathway [40], both of which act as cAMP-signaling effector proteins. Additionally, unlike in the study performed on bone-marrow MSCs [58], the heterozygous inactivating mutation of Gsα in murine ADSCs increased the level of both early and late osteogenesis markers in vitro. The expression of *RUNX2*, *SPP1*, *OCN*, and mineralization was increased in ADSCs obtained from mutant animals [28]. Therefore, generally, the effect of cAMP signaling seems to be different in ADSCs than that in BMSCs—in the case of ADSCs, increased signaling favors adipogenesis, while impaired signaling favors osteogenesis.

Results on the early osteogenesis of ADSCs obtained in this study support this hypothesis. Additionally, the upregulation of osteogenesis by FSK–IBMX at the stage when ADSCs were already differentiated into preosteoblasts agrees with the literature data. In BMSCs, which are more prone to osteogenesis than ADSCs [7,9,60], osteogenesis is upregulated by cAMP signaling. Nonetheless, it remains to be determined what the mechanisms of the cAMP inhibitory effect are on early osteogenic differentiation, and of the stimulatory effect on late osteogenic differentiation. Further studies should aim to determine how cAMP affects other osteogenesis-related signaling pathways, such as the ERK and canonical β-catenin pathways, during ADSC differentiation.

## 5. Conclusions

The biological outcome of cAMP level regulation depends on the current ADSCs’ differentiation stage. The upregulation of cAMP signaling can enhance osteogenesis if applied at the later stages of osteogenic differentiation. This observation may explain the discrepancy between the results of osteogenic differentiation obtained by other groups. Hence, cAMP level manipulation might be used to steer the differentiation of ADSCs into osteoblasts prior to implantation on 3D scaffolds in prospective bone-regeneration clinical studies.
